# Hierarchically porous, and Cu- and Zn-containing γ-AlOOH mesostrands as adjuvants for cancer immunotherapy

**DOI:** 10.1038/s41598-017-12446-9

**Published:** 2017-12-01

**Authors:** Xia Li, Mohamed A. Shenashen, Xiupeng Wang, Atsuo Ito, Akiyoshi Taniguchi, Sherif A. EI-Safty

**Affiliations:** 1 Green Recycling Process Group, Research Center for Functional Materials, National Institute for Materials Science, 1-2-1 Sengen, Tuskuba, Ibaraki 305-0047 Japan; 2 Human Technology Research Institute, National Institute of Advanced Industrial Science and Technology (AIST), Central 6, 1-1-1 Higashi, Tsukuba, Ibaraki 305-8566 Japan; 3 Cellular Functional Nanomaterials Group, Research Center for Functional Materials, National Institute for Materials Science, 1-1 Namiki, Tsukuba, Ibaraki 305-0044 Japan; 4 Faculty of Engineering and Advanced Manufacturing, University of Sunderland, St Peter’s Campus, St Peter’s Way, Sunderland, SR6 0DD UK

## Abstract

Alum is the only licensed adjuvant by Food and Drug Administration of USA used in many human vaccines and has excellent safety record in clinical applications. However, alum hardly induces T helper 1 (Th1) immune responses that are required for anti-tumor immunity. In the present study, we fabricated hierarchical copper- and zinc- buds dressing γ-AlOOH mesostrands (Cu- and Zn-AMSs) with randomly wrinkled morphology, mesoscale void- or cave-like pockets, high-exposed surface coverage sites, and positive charge streams in saline. We confirmed that Cu- and Zn-AMSs promoted intracellular uptake of model cancer antigen (ovalbumin, OVA) by THP-1-differentiated macrophage-like cells *in vitro*. Moreover, Cu- and Zn-AMSs enhanced maturation and cytokine release of bone marrow dendritic cells *in vitro*. *In vivo* study demonstrated that Cu- and Zn-AMSs markedly induced anti-tumor-immunity and enhanced CD_4_^+^ and CD_8_^+^ T cell populations in splenocytes of mice. These findings demonstrated that hierarchical copper- and zinc- buds dressing γ-AlOOH mesostrands, which are oriented in randomly wrinkled matrice, are suitable platforms as novel adjuvants for cancer immunotherapy.

## Introduction

Recently, cancer immunotherapy has greatly increased its clinical benefit, because it is able to remove inhibitory pathways or enhance stimulatory pathways in the immune system to eradicate cancer cells^1–6^. The inhibitory pathways in tumor microenvironments include expression and/or release of immunosuppressive molecules from tumor cells, and the poor immunogenicity of tumor antigen that inhibits recognition and elimination of the tumor cells by the immune system^1–6^. To alter the immunosuppressive tumor microenvironments and/or strengthen immunogenicity of tumor antigen, suitable immunoadjuvant need be introduced to boost immune system and to initiate anti-tumor immune response^2,3,7–9^.

Alum is the only adjuvant approved by US Food and Drug Administration (FDA) in human vaccines such as human papillomavirus vaccine and hepatitis B vaccine^10^. Alum has excellent safety record in clinical use^11,12^, since its first discovery in 1926 as the adjuvant in diphtheria toxin vaccine^13^. General mechanism of alum is believed to be as the depot for delayed clearing of antigen and facilitating uptake by antigen-presenting cells (APCs)^14^. In addition, other hypothetical mechanisms were proposed about alum; such mechanisms include activating intracellular innate immune response system^15^, triggering dendritic cells response by altering membrane lipid structures^16^, and causing DNA release from dying host cells^17^. Although conventional alum is widely used and quite effective in human vaccines for infectious diseases, its efficacy for cancer vaccines is ambiguous. Alum is generally recognized to strongly affect promotion of T helper (Th) proteins and Th2 immune response and weakly influence Th1 immune response. However, for anti-tumor immune response, Th1 immune response plays more important roles than Th2. Interestingly, recent studies showed that Th1 immune response may be strengthened by combination of some biomolecular components with alum or α-Al_2_O_3_ nanoparticles. For example, interleukin (IL)-12–aluminum adjuvant complex can enhance Th1 immune response, and this action can be further augmented with coexistence of IL-18^18,19^. In another case, α-Al_2_O_3_ nanoparticles enhanced antigen cross-presentation, activated OT-I CD_8_^+^ T cells and significantly improved antitumor immune response^20^. Although clinical use of IL-12 and α-Al_2_O_3_ nanoparticles would be challenging due to potential toxicity, high-cost associated with the former and poor degradability associated with the latter, these examples motivated us to alter chemistry and structure of alum adjuvant to strengthen Th1 immune response and to enhance its adjuvanticity in anti-tumor immunity.

Recently, efforts were focused on creating novel hierarchical nanocrystals with unique features for different potential field applications, including biomedicine or catalysis^21,22^. Since discovery of mesoporous metal oxides, several methods were developed for fabrication of hierarchical mesocrystals with controlled pore spaces, shapes, and highly exposed surface sites^23,24^. Researchers have particular interest in wide applications of aluminum oxohydroxides, such as boehmite (γ-AlOOH) nanocrystals, with Lewis acidity, surface morphologies, and geometries, such as sheets, plates, and flakes, which can be controlled under wide-range synthesis conditions^25–28^. Boehmite structures are mainly organized in stacked, octahedral layers of Al(OOH)_6_ units through shared edges connected by hydrogen bonds^29^.

Trace elements copper and zinc are essential for growth of all organisms and affect development and integrity of immune system. Copper is essential for maintenance of immune function and activities of specific immunological markers^30,31^. Zinc has broad influence on key immunity mediators, such as enzymes, thymic peptides, and cytokines; proliferation and differentiation of immune cells necessitates constant supply of sufficient amounts of zinc^32,33^. Zinc deficiency may result in reduced activity of thymus and thymic hormones, phagocytosis, T-helper cell numbers, natural killer cell activity, antibody production and cell-mediated immunity, and shift of Th1 cell balance toward Th2 cells^34,35^.

**Figure 1:**
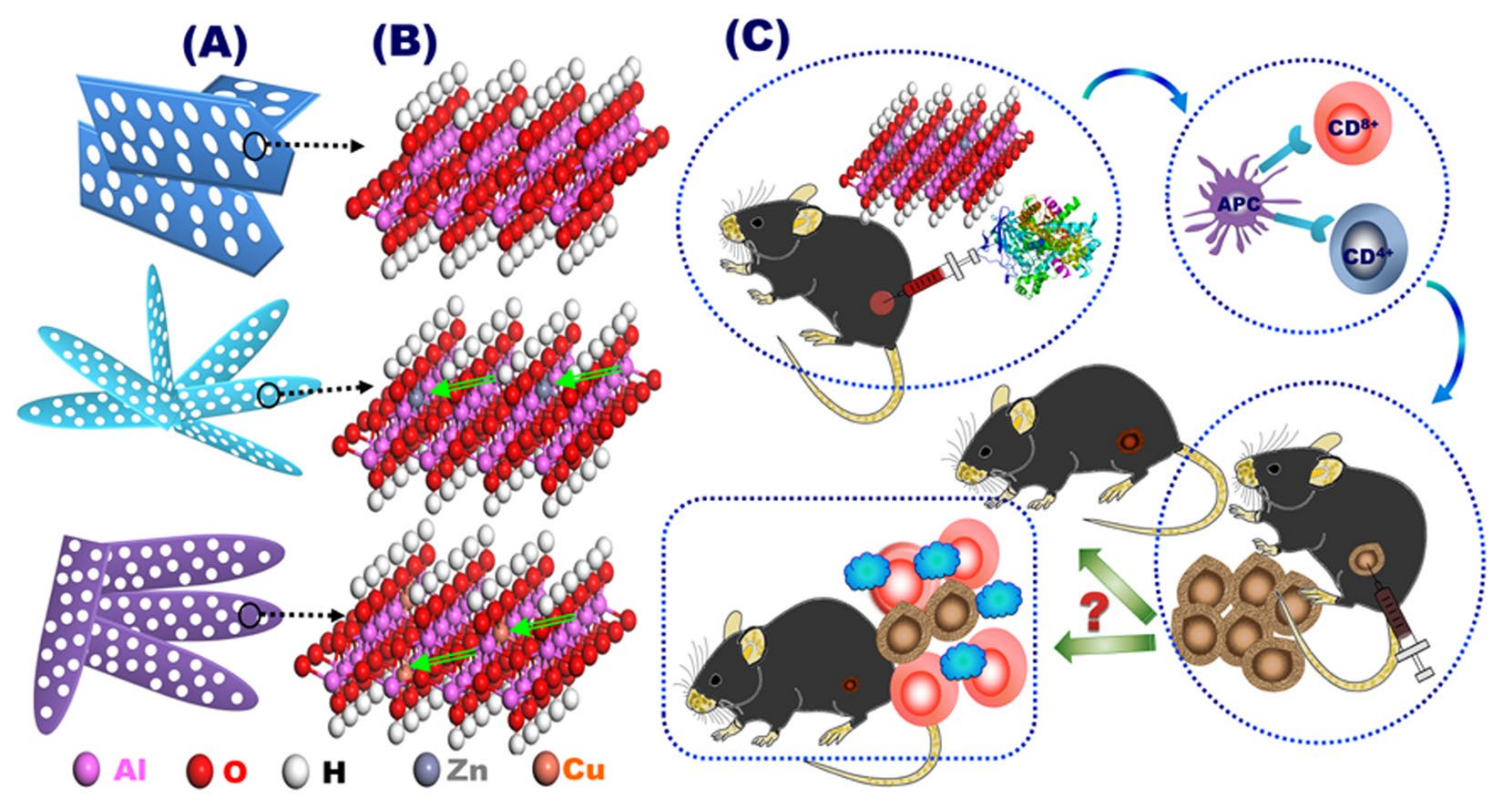
Hierarchical Cu- and Zn-buds dressing γ-AlOOH mesostrands that act as immunoadjuvants for stimulating anti-tumor immune response. (**A**,**B**) mesoscale atomic arrangements of hierarchical Cu- and Zn-buds dressing γ-AlOOH mesostrands morphology (**A**) and crystal structures (**B**). (**C**) The method for activation of anti-tumor immune response as follows: (i) immunization of mouse with the Cu-, and Zn-AMSs-OVA complex; (ii) antigen-presenting cells accumulation and antigen presentation to T cells; (iii) subsequent injection of EG7-OVA cancer cells; and iv) checking test of whether the tumor growth was inhibited.

In the present study, we hierarchically engineered copper- and zinc- buds dressing γ-AlOOH mesostrands with randomly wrinkled matrice, oriented vascular channels, mesoscale void- or cave-like pockets, interior decoration of metal ion buds and positive charge surface streams, acting as immuoadjuvant agents to enhance anti-tumor immunity. Cu- and Zn-AMSs immunoadjuvants enhanced cellular uptake of cancer model antigens into THP-1-differentiated macrophage-like cells, enhanced population of CD_4_^+^ and CD_8_^+^ T cells, promoted secretion of Th1 cytokines, evoked strong Th1 immune response, and inhibited EG7-OVA tumor growth (Fig. 1) compared with that of pristine γ-AlOOH mesostrands. On mesoscale atomic arrangement, the developed hierarchical metal ion-buds dressing γ-AlOOH mesostrands can serve as novel immunoadjuvants compared with currently alum adjuvant for cancer vaccines.

## Results

### Characterization of hierarchical copper- and zinc- buds dressing γ-AlOOH mesostrands

**Figure 2:**
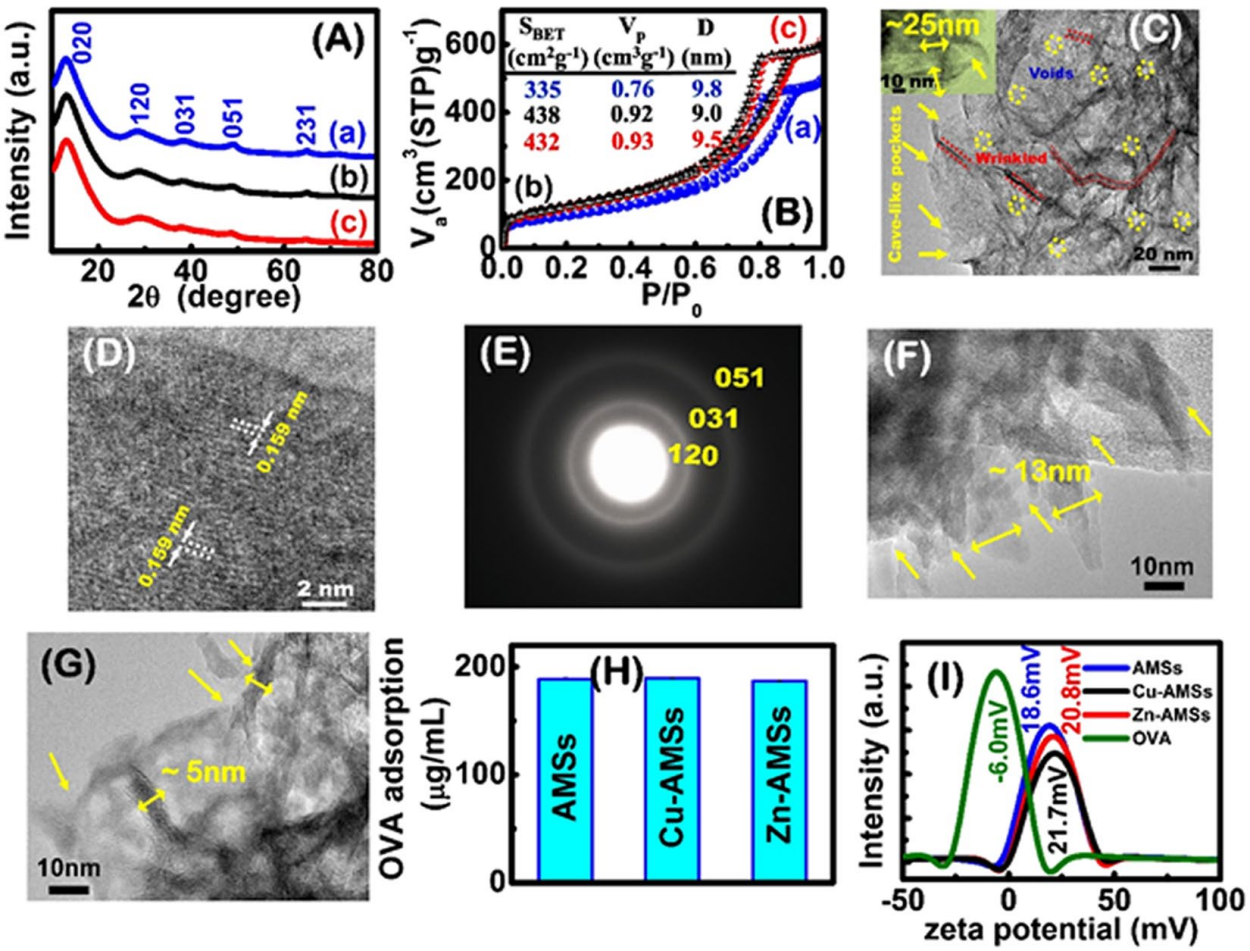
(**A**) Wide-angle X-ray diffraction (WAXRD) and (**B**) N_2_ sorption isotherms and pore structure parameters (insert one) of hierarchical AMSs (a), Cu-AMSs (b) and Zn-AMSs (c). (**C–E**) TEM images (**C**), high-magnification TEM (HRTEM) image (**D**), and electron diffraction (**E**) of AMSs. (**F**,**G**) TEM images of Cu-AMSs (**F**) and Zn-AMSs **(G**), respectively. (**H**) OVA adsorption onto different AMSs samples. (**I**) Zeta potential of OVA and hierarchical AMSs, Cu-AMSs and Zn-AMSs.

We successfully fabricated hierarchical Cu- and Zn-AMSs mesostrands with randomly wrinkled morphology (Fig. 2). Pristine γ-AlOOH mesostrands (AMS) were also prepared. The wide-angle X-ray diffraction peaks of all the samples were indexed without difficulty as the orthorhombic γ-AlOOH with lattice parameters of a = 3.691 Å, b = 12.24 Å, and c = 2.859 Å (JCPDS no. 21-1307). There is no obvious difference between the pristine AMSs, Cu-AMSs, and Zn-AMSs samples, indicating the formation of copper- and zinc- substituted γ-AlOOH structure. FTIR spectra further suggest the formation of γ-AlOOH based material (Supplementary Figure 1). The three characteristics absorption bands at 486, 623, and 751 cm^−1^ correspond to the formation of Al–O bonds of the boehmite AlO_6_ octahedrons. The characteristic absorption bands at 1067 cm^−1^ is assigned to Al–O–H and the two absorption bands at 3090 and 3392 cm^−1^ are assigned to the stretching vibration for the symmetrical and asymmetrical modes of the O–H bonds that are attached to the oxyhydroxide surface. Figure 1B shows double layers of AlO_4_(OH)_2_ with aluminum octahedral unit center connected to six atoms of oxygen; four of these coordination bonds include oxides, and remaining two have hydroxide on surfaces. The pristine AMSs, Cu-AMSs, and Zn-AMSs samples exhibit a type IV isotherm with H_2_-type hysteresis loop, which is characteristic of mesoporous materials. A high surface area of approximately 335 m^2^/g, 438 m^2^/g and 432 m^2^/g, a high pore volume of approximately 0.76, 0.92 and 0.93 cm^3^/g, and a narrow pore distribution of approximately 9.8, 9.0 and 9.5 nm are obtained for AMSs, Cu-AMSs and Zn-AMSs, respectively (Fig. 2B). The specific surface area and the total pore volume increased with the presence of Cu and Zn. The mesoporosity of the material can be attributed to the pores formed as a result of aggregated γ-AlOOH and the void space in the layer of the sample. N_2_ adsorption–desorption isotherms indicate that pristine AMSs, Cu-AMSs, and Zn-AMSs samples had large surface-area-to-volume ratios, mesoscale void- or cave-like pockets. Transmission electron microscopy (TEM) images show the formation of distinct mat-like patterns consisted of branched and wrinkled tubes of γ-AlOOH mesostrands with length of 80~100 nm and width of 25 nm (Fig. 2C). Along these wrinkled nets, mat patterns show dispersed mesoscale void spaces and spherical caves formed by interlinkage of random-branches. The image displays formation of window cavities and humped protuberance of needle like tubes along surface edges of mesostrands. Well-developed lattice planes further demonstrate crystallinity with interplanar spacing of 0.159 nm, describing crystal lattice plane (220) (Fig. 2D). Selective area electron diffraction pattern shows ring-like shapes of abundant resolutions of (120), (031), and (051) fringes of crystalline mesostrands, as shown in Fig. 2E. The copper dressing mesoporous aluminum oxyhydroxide nanostrands exhibit the strand morphology with length of 40~80 nm and width of 13 nm (Fig. 2F). While, the dressing of zinc in mesoporous aluminum oxyhydroxide nanostrands resulted the decrease in the strand size with length of 30~50 nm and width of 5 nm (Fig. 2G). The dressing of copper and zinc in mesoporous aluminum oxyhydroxide inhibited the growth of strand size. Cu- and Zn-AMSs clearly attained hierarchal formation of voids, humped protuberance on surface, and mesostrands channels. The hydrodynamic size of AMSs, Cu-AMSs and Zn-AMSs in PBS buffer is 314.9 ± 69.5 nm, 271.4 ± 72.1 nm and 248.7 ± 62.2 nm, respectively (Supplementary Figure S2). The hydrodynamic size of the samples is higher than that observed in the microscopic TEM patterns because of the particle aggregation in PBS solution. The AMSs, Cu-AMSs, and Zn-AMSs samples adsorb a high amount of ovalbumin (OVA) (Fig. 2H) due to positive charge associated with these mesostrands and negative charge associated with OVA in saline (Fig. 2I). Moreover, the high surface area of hierarchical mesostrand structure of AMSs, Cu-AMSs and Zn-AMSs samples facilitates the adsorption of OVA onto the carriers. The hierarchical copper- and zinc- buds dressing γ-AlOOH mesostrands exhibited (i) the interior formation of metal ions buds in the mesostrands skeleton, (ii) mesoscale void- or cave-like pockets oriented along whole mat-like tangles, (iii) randomly wrinkled morphology, (iv) high-exposed surface coverage sites, (v) positive charge streams in saline.

### Cellular uptake of model antigen - loaded mesostrands by THP-1-differentiated macrophage-like cells

**Figure 3:**
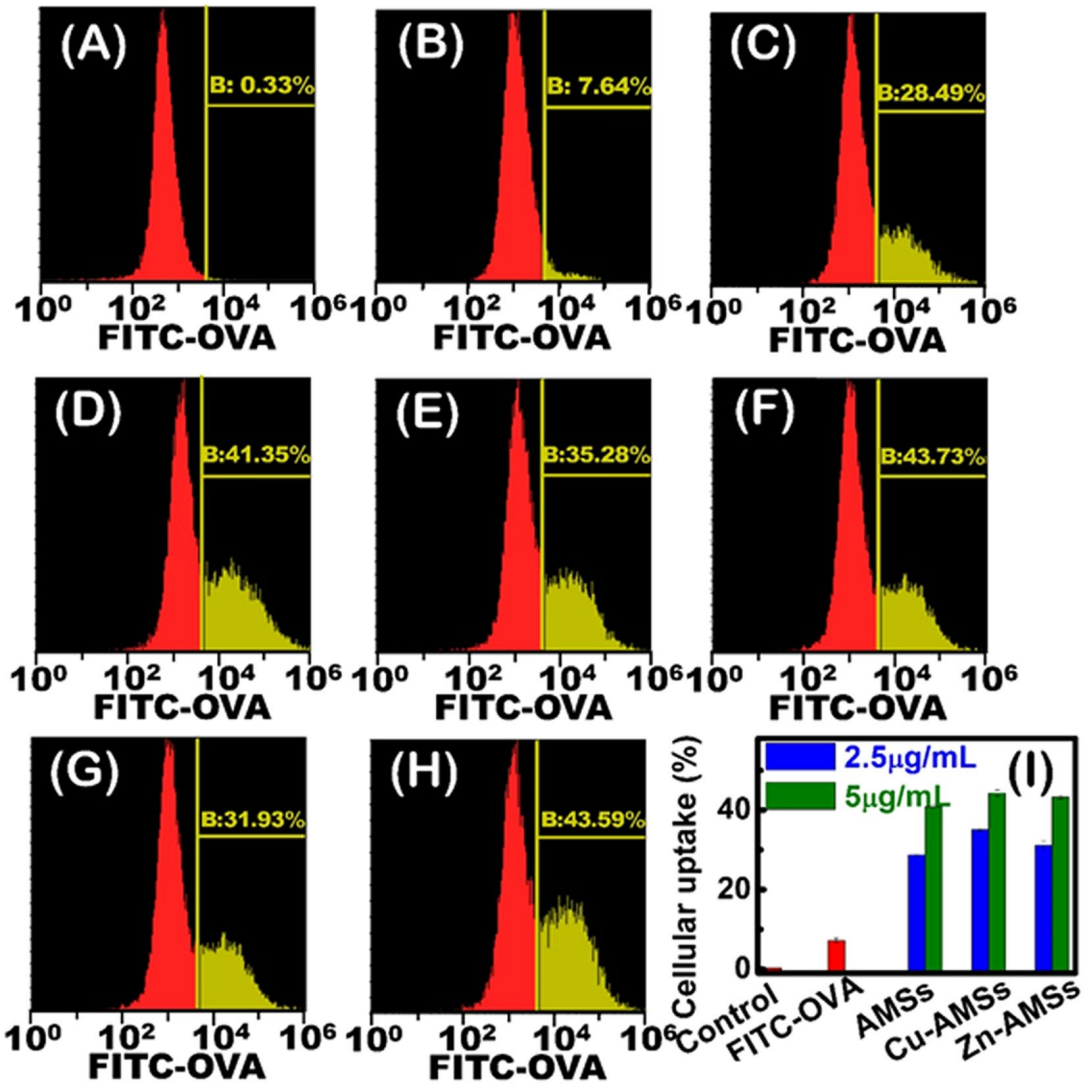
Cellular uptake of FITC-OVA by THP-1 differentiated macrophages. (**A**) Histograms for control, (**B**) soluble FITC-OVA, (**C**,**D**) FITC-OVA-AMSs (particle concentration: (**C**), 2.5 μg/mL; (**D**) 5 μg/mL), (**E**,**F**) FITC-OVA-Cu-AMSs (particle concentration: (**E**) 2.5 μg/mL; (**F**) 5 μg/mL) and (**G**,**H**) FITC-OVA-Zn-AMSs (particle concentration: (**G**) 2.5 μg/mL; (**H**) 5 μg/mL). (**I**) Semiquantatative analysis for control, soluble FITC-OVA, FITC-OVA-AMSs, FITC-OVA-Cu-AMSs and FITC-OVA-Zn-AMSs at different particles concentration (2.5 and 5 μg/mL).

**Figure 4:**
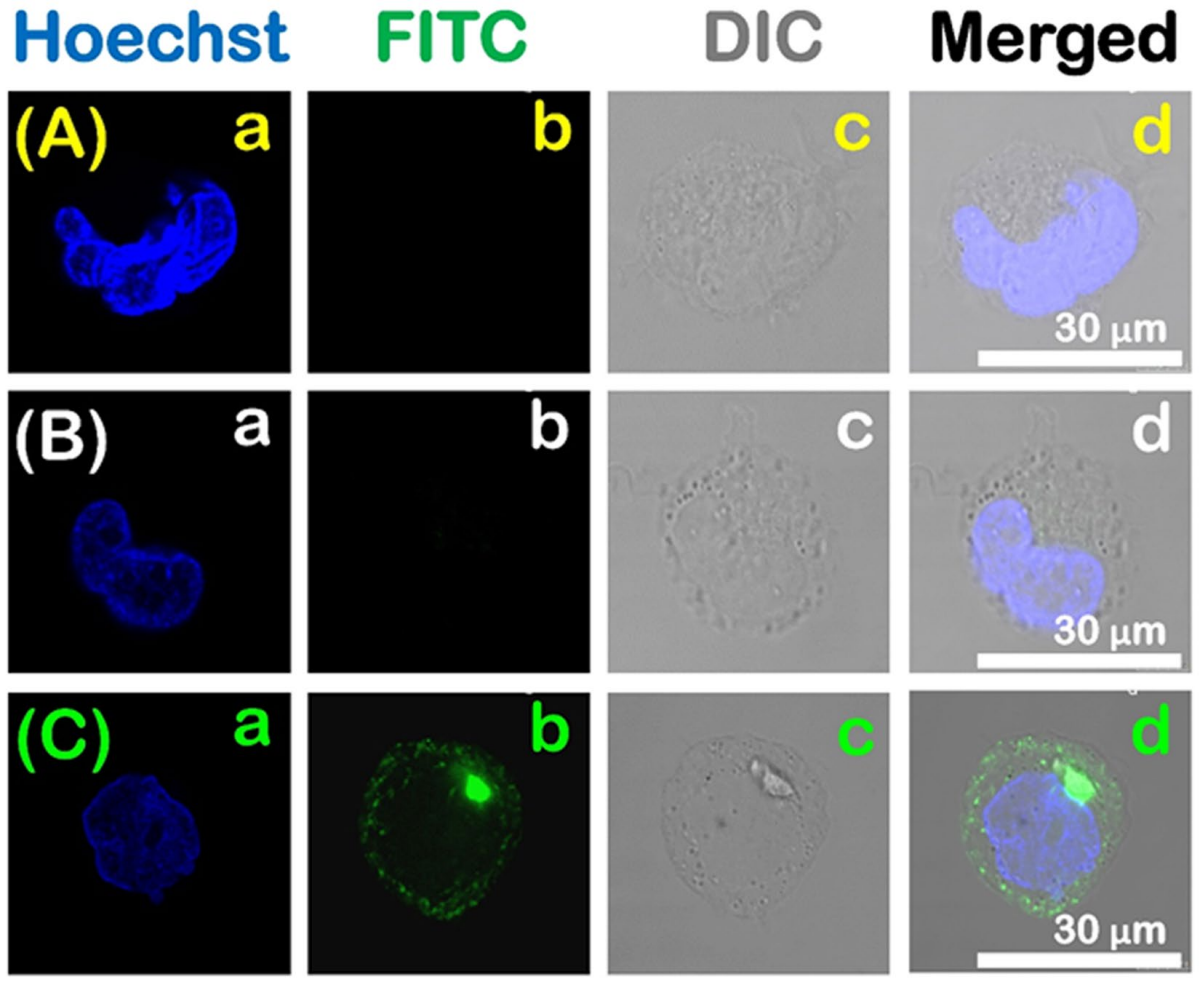
Confocal laser scanning microscopy images of the cellular uptake by THP-1 differentiated macrophages: the control (**A**), soluble FITC-OVA (**B**) and FITC-OVA-Cu-AMSs (**C**) samples.

Since cellular uptake of antigen by antigen-presenting cells is the first step of adaptive immune responses, cellular uptake of fluorescent model antigen (FITC-OVA) was tested by using macrophage-like cells differentiated from THP-1 monocytes (Fig. 3). FITC-OVA was loaded onto hierarchical AMSs, Cu-AMSs, and Zn-AMSs with random wrinkle and channels. The loading FITC-OVA on mesostrands efficiently increased cellular uptake of OVA by THP-1-differentiated macrophage-like cells. For example, macrophage-like cells-cultured with soluble FITC-OVA, FITC-OVA-AMSs, FITC-OVA-Cu-AMSs, and FITC-OVA-Zn-AMSs show positive cell ratios of 7.2% ± 0.6%, 28.8% ± 0.4%, 35.1% ± 0.3%, and 31.2% ± 1.1%, respectively. In addition, with increasing particle concentration in culture medium, ratio of FITC-OVA positive cells increases. Using confocal laser scanning microscopy (CLSM), we observed directly cellular uptake of FITC-OVA into THP-1-differentiated macrophage-like cells (Fig. 4). The macrophage-like cells cultured with soluble FITC-OVA only show weak green fluorescence. The cells cultured with FITC-OVA-Cu-AMSs exhibit significantly enhanced green fluorescence.

### Maturation and cytokine release of bone marrow dendritic cells (BMDCs)

The hierarchical AMSs, Cu-AMSs, and Zn-AMSs - containing medium were cultured with BMDCs to investigate the effects of mesostrands on maturation and cytokine release of dendritic cells (Supplementary Figure 3). The medium without any particles was used as control. The CD86^+^CD11c^+^ cell populations of control, AMSs, Cu-AMSs and Zn-AMSs, representing early dendritic cells maturation, are 10.4 ± 1.6%, 11.4 ± 1.6%, 13.9 ± 2.4% and 13.8 ± 2.0%, respectively. Zn-AMSs significantly promote the maturation of BMDCs compared with control. In addition, Cu-AMSs and Zn-AMSs significantly enhance the secretion of interferon-γ (INF-γ) cytokine from BMDCs.

### Anti-tumor efficacy of mesostrands in EG7-OVA lymphoma model

**Figure 5:**
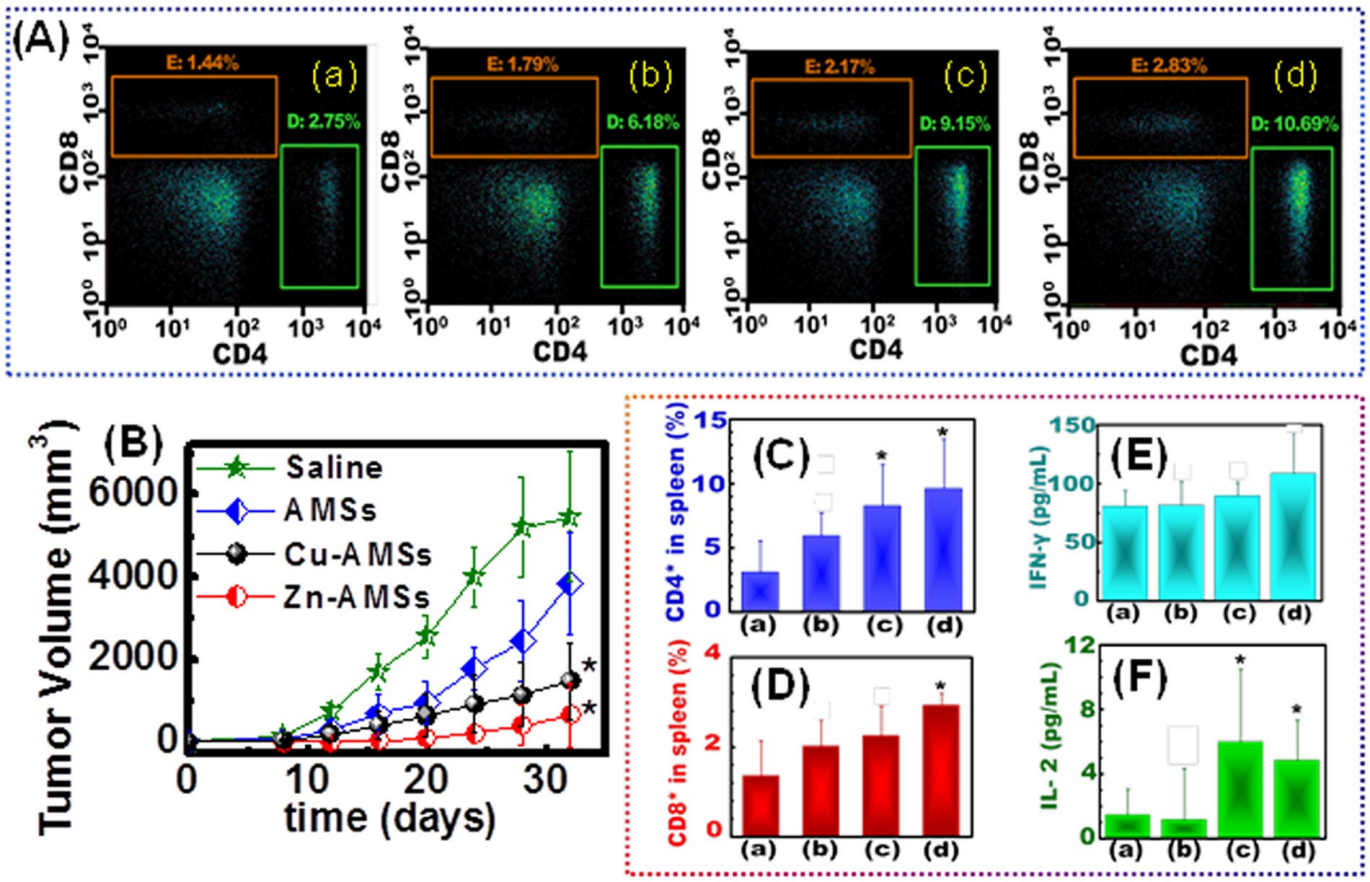
Hierarchical Cu-AMSs and Zn-AMSs induce anti-tumor immunity in an EG7-OVA lymphoma model. (**A**) The CD4 and CD8 cells populations in spleen of mice at the endpoint for control (a), OVA-AMSs (b), OVA-Cu-AMSs (c), OVA-Zn-AMSs (d). (**B**) tumor volume of mouse immunized with OVA-saline, OVA-AMSs, OVA-Cu-AMSs and OVA-Zn-AMSs 32 days after EG-7. OVA cancer cells injection. (**C**,**D**) Statistic analysis of CD4 (**C**) and CD8 (**D**) cell population in splenocytes. (**E**,**F**) Cytokines content in spleen of mice at the endpoint for control (a), OVA-AMSs (b), OVA-Cu-AMSs (c), OVA-Zn-AMSs (d).

To check adjuvanticity in inducing anti-tumor immunity, C57BL/6 mice were immunized with the model tumor antigen OVA with and without the hierarchical mesostrands followed by the challenge with subcutaneous injection of EG7-OVA lymphoma cells *in vivo* (Fig. 5B). Immunization by OVA with hierarchical mesostrands all suppress tumor growth compared with control without mesostrands. Mice immunized with OVA-Cu-AMSs or OVA-Zn-AMSs exhibit a tendency of a higher degree of suppression of tumor growth compared with those immunized with OVA-AMSs. OVA-Zn-AMSs exhibit optimum anti-tumor efficacy compared with other mesostrands. Finally, the tumor size for control, OVA-AMSs, OVA-Cu-AMSs, and OVA-Zn-AMSs are 5471 ± 1582, 3853 ± 1234, 1476 ± 930 mm^3^, and 650 ± 783, respectively, 32 days after the challenge.

### CD_4_^+^ and CD_8_^+^ T cell populations, Th1 cytokine secretion and lymph node metastasis

To further investigate mechanism of anti-tumor immune response, we analyzed CD_4_^+^ and CD_8_^+^ T lymphocyte population and production of INF-γ and IL-2 in spleen (Fig. 5A,C–F). When Cu-AMSs and Zn-AMSs were used as adjuvants, significant enhancement was noted in CD_4_^+^ and CD_8_^+^ population in spleen compared with saline group. CD_4_^+^ T cell populations in spleen of mice immunized with OVA-saline, OVA-AMSs, OVA-Cu-AMSs and OVA-Zn-AMSs are 3.1% ± 2.5%, 5.9% ± 1.8%, 8.3% ± 3.2%, and 9.6% ± 3.8%, respectively. CD_8_^+^ T cell populations in spleen of mice treated with OVA-saline, OVA-AMSs, OVA-Cu-AMSs, and OVA-Zn-AMSs are 1.4% ± 0.8%, 2.0% ± 0.6%, 2.3% ± 0.7%, and 2.9% ± 0.3%, respectively. Mice treated with OVA-Zn-AMSs have the highest average level of INF-γ. For IL-2, mice immunized with OVA-Cu-AMSs and OVA-Zn-AMSs show high contents compared with control and OVA-AMSs groups. Histological sections of lymph node for mice immunized with OVA-saline and OVA-Zn-AMSs were carried out to check the metastasis of cancer (Supplementary Figure S4). In the histological section for OVA-saline, many abnormal cancer cells with irregular size and random shape occur. However, for OVA-Zn-AMSs, no obvious irregular cells were observed and large amounts of immune cells are present.

## Discussion

A key issue for cancer immunotherapy is to overcome immune tolerance that includes tumor induced immunosuppression and escape in the tumor microenvironment. Although recent successes in clinic demonstrated great potential in cancer immunotherapy, significant work must still be accomplished to modulate immune system to achieve satisfactory therapeutic efficacy at reasonable expense. To enhance therapeutic effectiveness of cancer immunotherapy, promising strategies involve improvement of adjuvants for cancer vaccines. Alum is the only approved adjuvant by FDA in many human vaccines. However, alum adjuvant tends to induce Th2 immunity and is weakly effective for Th1 immunity. Herein, we introduced novel metal-ion-bud dressing randomly wrinkled AMS as effective adjuvants, which enhanced cellular uptake of model cancer antigen by APCs, increased CD_4_^+^ and CD_8_^+^ T cell populations, increased Th1 cytokine contents in spleen, favored Th1 anti-tumor immune response, and thus inhibited tumor growth in the mice model.

Copper and zinc were facilely incorporated into γ-AlOOH mesostrands via one-pot synthesis pathway to fabricate hierarchically designed, metal-ion-bud dressed and randomly wrinkled mesostrand mats having mesoscale void space, spherical caves, and mesostrand channels. Presence of copper or zinc in synthesized solution significantly reduced the particle size of γ-AlOOH mesostrand. Similar phenomenon was also observed in the formation of hydroxyapatite with presence of copper or zinc; obtained copper- or zinc- doped hydroxyapatite exhibited much smaller particle size compared with pure one^36,37^.

With or without dressing of copper or zinc, hierarchically engineered and randomly wrinkled mesostrand mats significantly promoted cellular uptake of model antigen by APCs compared with the soluble antigen. Cellular uptake of antigen by APCs is the first step to initiating adaptive immune responses. Improvements were observed on cellular uptake of antigens by APCs and bioavailability of antigens. These results were caused by the presence of hierarchically engineered and randomly wrinkled mesostrand mats with large surface-area-to-volume ratios, mesoscale void- or cave-like pockets. Co-administration of randomly wrinkled mesostrands with antigens served as depot for effective antigen delivery system and increased possibility of sustained release of antigens. Prolonged antigen exposure possibly facilitated activation of antigen-presenting cells, presentation of antigens to T cells, and improvement of antitumor immune response. Subsequent experimental data included CD_4_^+^ and CD_8_^+^ T cells ratio, Th1 cytokine contents in spleen, and anti-tumor efficacy and provided evidence at this point.

Bioelements copper and zinc reportedly enhanced host immune system. Copper and zinc are essential trace elements and affect multiple aspects of innate and adaptive immunity^30–33^. Copper-containing enzymes include Cu–Zn-superoxide dismutase, cytochrome c oxidase, and diamine oxidase^31^. Copper deficiency caused reduction in number of neutrophils in human peripheral blood, IL-2 production, and T cell proliferation^30,31^. In male rats, Cu deficiency resulted in decreased total splenic mononuclear cell yield, including relative percentage and absolute number of T-cells and number of CD_4_^+^ helper and CD_8_^+^ cytotoxic T-subsets^38^. Two-month Cu supplementation with 10 mg/day induced increased IL-2 in healthy adults^39^. Zinc is cofactor in more than 300 enzymes influencing various organ functions^32^. Sufficient amounts of zinc is necessary for proliferation and differentiation of immune cells^32,33^, and zinc supplement can promote activity of APCs and Th1 cell responses. Zinc deficiency may result in decreased phagocytosis, T-helper cell numbers, natural killer cell activity, antibody production, and cell-mediated immunity^34,35^. Zinc can also affect Th1/Th2 balance. For instance, zinc deficiency reduced production of Th1 cytokines, in particular IFN-γ, IL-2, and tumor necrosis factor (TNF)-α, whereas levels of Th2 cytokines IL-4, IL-6, and IL-10 were not affected^40,41^.

In spleen, CD_4_^+^ and CD_8_^+^ T cell ratios were increased by hierarchical metal ions-buds dressing mesostrands in wide domain-like randomly wrinkled mats. Antitumor immune regulation relies mainly on CD_4_^+^ helper T cells and CD_8_^+^ cytotoxic T cells. Much attention was devoted to role of CD_8_^+^ T cells in antitumor immunity^42^. CD_8_^+^ T cells express T-cell receptors, recognize antigenic peptides presented by major histocompatibility complex (MHC) class I molecules, and directly kill target cells expressing specific antigens. CD_4_^+^ T cells are considered to play important role in antitumor immunity by regulating differentiation and development of CD_8_^+^ T cells^43,44^. CD_4_^+^ T cells recognize antigenic peptides presented by MHC class II molecules and release vast variety of Th1 or Th2 cytokines to regulate immune responses, which facilitate activation of CD_8_^+^ T cells or stimulation of B cells for antibody production^43,44^. In this study, hierarchical metal ions-buds dressing mesostrands significantly promoted CD_4_^+^ and CD_8_^+^ T cell populations in spleen through enhanced antigen presentation by APCs. Hierarchical metal ion-buds dressing in randomly wrinkled mesostrand mats led to increased secretion of Th1 cytokines and enhanced Th1 immunity. In immune system, several different cell types communicate with one another by exchanging cytokines as cell signal to coordinate appropriate immune response. Th1 cytokines include IFN-γ, IL-2, and TNF-α, whereas Th2 cytokines include IL-4, IL-5, IL-6, IL-10, etc. IFN-γ, which is major product of Th1 cells, is crucial for strengthening innate and adaptive immune responses against tumor and further skews immune response toward Th1 phenotype^45^. IFN-γ is secreted predominantly by natural killer cells as part of innate immune response and by CD_4_ and CD_8_ T lymphocytes once adaptive immune responses develops^46^. IFN-γ upregulates both MHC classes I and II antigen presentation, contributes to macrophage activation, enhances lymphocyte recruitment, prolongs lymphocyte activation in tissues, and promotes differentiation of naive CD_4_ T cells into Th1 effectors^46^. IL-2 is an important T cell growth factor, which triggers immune system to produce T cells, promotes progression of T-lymphocytes from G1 phase to S phase of cell cycle, and helps development of T cell immunological memory^47–49^. High doses of IL-2, either alone or combined with other cancer vaccines, can mediate anti-tumor immunity and was studied extensively as cancer therapeutic cytokines in clinical trials; however, its toxicity limited its therapeutic benefits^47^. IL-2 enhanced antitumor activity of HLA-A0201-restricted modified gp100 209 2 M peptide vaccine^50^. In detail, eight out of 19 patients (42%) receiving peptide plus IL-2 had objective cancer regressions, whereas none of 11 patients receiving peptide alone showed the same result^50^. In other clinical trials, 6% of 270 patients with metastatic melanoma achieved complete remissions when administered with high-dose of IL-12^51^. This work used hierarchical metal ions-buds dressing mesostrands; material observed stimulated secretion of Th1 cytokines, such as IFN-γ and IL-2, to enhance anti-tumor immune response. Interestingly, in contrast to alum, α-Al_2_O_3_ conjugated with OVA was reported to greatly improve antitumor efficacy and stimulate T cells to release both IFN-γ and IL-2^20^. Developed hierarchical metal ions-buds dressing mesostrands in wide domain-like randomly wrinkled mats are key point to stimulating secretion of Th1 cytokines, promoting Th1 immunity, and inhibiting EG7-OVA tumor growth.

In conclusion, we fabricated hierarchical metal ions-buds dressing mesostrands in wide domain-like randomly wrinkled mats. The mesostrands markedly enhanced cellular uptake of a model cancer antigen, OVA, in APCs. Our results showed that these mesostrands are valuable as immunoadjuvants for cancer immunotherapy since the mesostrands evoke strong Th1 immune response and anti-cancer immunity, increase CD_4_^+^ and CD_8_^+^ T cell populations in splenocytes of mice, and inhibit EG7-OVA tumor growth. Our results provide strong evidence that developed hierarchical copper- and zinc- buds dressing γ-AlOOH mesostrands may open new gate of enhancing anti-tumor immunity.

## Methods

### Materials synthesis

Hierarchically engineered copper- and zinc- buds dressing γ-AlOOH mesostrands were synthesized by using inorganic aluminum salts (AlCl_3_) as Al sources, CuCl_2_·2H_2_O (or ZnCl_2_) as Cu (Zn) sources, and triblock copolymer P123 as template (see Supporting Information). In typical synthesis, 0.00016 mol of P123, 0.009 mol of AlCl_3_·6H_2_O, and 0.001 mol of CuCl_2_·2H_2_O (or ZnCl_2_) were mixed in 13.4 ml water and aged in shaking bath at 40 °C for one day. Then, ammonia was added to the above sol solution to adjust the pH to 8 and hydrothermally treated at 60 °C for 24 h. Control included prepared materials without adding CuCl_2_·2H_2_O (or ZnCl_2_). Final products were collected by centrifuge, extracted in ethanol for 24 h, and washed with water and ethanol thrice.

### Loading of model antigen (OVA) onto carriers

A total of 3 mg of particles were mixed with OVA solution at 0.2 mg/mL in saline and shook at 1000 rpm overnight at room temperature. Then, mixture was centrifuged, and supernatant was collected to test loading amount.

### Cellular uptake assay of OVA by macrophage-like cells

To determine cellular uptake of model antigen OVA, THP-1 monocytes were differentiated to obtain macrophage-like cells. In detail, 2 × 10^6^ cells·mL^−1^ of THP-1 cells were differentiated by culturing in RPMI1640 medium supplemented with 3% fetal bovine serum and 800 nM of phorbol 12-myristate 13-acetate (PMA) for four days. Then, cells were washed with phosphate-buffered saline (PBS) _(−)_ and cultured with fresh medium without PMA for another one day. Copper- and zinc- doped mesoporous γ-AlOOH were dispersed in saline by ultrasonication and mixed with FITC-OVA overnight at room temperature. FITC-OVA-loaded particles were added into culture medium with final concentration of 2.5 or 5 µg/mL for particles and 5 µg/mL for FITC-OVA. FITC-OVA was used as control at the same dose. After culturing for 4 h, cells were washed with PBS _(−)_ twice, trypsinized, and collected for cellular uptake test by using spectral cell analyzer (Sony, LE-SP6800A).

To directly observe cellular uptake of model antigen, FITC-OVA loaded particles were cultured with THP-1-differentiated macrophage-like cells. After incubation cells for 8 h, cells were washed with PBS twice and fixed with 4% paraformaldehyde for 20 min. Then, cell nucleus was stained with Hoechst. Cellular uptake of complexes was visualized by CLSM (SP5, Leica).

### Maturation and cytokine release of dendritic cells

To evaluate the adjuvant property, the maturation of dendritic cells and the cytokine release of IFN-γ were quantified by using bone marrow dendritic cells (BMDCs). In detail, bone marrow cells were harvested from femurs of mice, followed with red blood cell lysis. Subsequently, the residual cells were labeled with phycoerythrin-conjugated anti-CD4, CD8, and I-A/I-E (eBioscience), and depleted by anti-phycoerythrin magnetic beads (Miltenyi Biotec) and Auto MACS (Miltenyi Biotec). The obtained cells were cultured in RPMI 1640 medium containing 10% fetal bovine serum and 20 ng/mL granulocyte macrophage colony-stimulating factor (GM-CSF, Bioreagent). After nine days, nonadherent and loosely adherent cells were harvested. The assessment by staining with fluorescein isothiocyanate-conjugated CD11c (eBioscience) showed that the obtained cells purity was 80~85%.

The obtained BMDCs (2 × 10^5^ cells/well) were cultured in RPMI 1640 media with particles (20 µg/mL). The media were analyzed 24 hours later for INF-γ concentrations using mouse ELISA kits (BD Pharmingen) according to the manufacturer’s instructions. Then, the cells was washed with PBS_(−)_ containing 0.5% bovine serum albumin (Gibco), blocked with anti-CD16/CD32 antibody (2.4G2, BD Pharmingen) to prevent non-specific staining, stained with anti-mouse CD86 and anti-mouse CD11c antibodies (Biolegend) for 30 min and analyzed by FACSAria (BD Bioscience).

### *In vivo* anti-tumor immune response

Six-week female C57BL/6 J mice (Charles River, Japan) were used for *in vivo* evaluation. A mixture of OVA with AMSs, Cu-AMSs, and Zn-AMSs or without any adjuvant in 100 µL of saline was administered subcutaneously into left flank of mice for 1st vaccination. One group administered with only OVA in saline were used as control. Four and 10 days later, the same amount of particle suspensions without OVA were administered at the same site for 2nd and 3rd vaccination, respectively. Fourteen days later, a total amount of 5 × 10^5^ EG7-OVA cancer cells were administered subcutaneously into right flank of mice. Tumor diameter was measured using calipers at certain time interval. All animal experiments were permitted by the Ethical Committee of the National Institute for Materials Science (NIMS), Japan. All animal experiments and feeding were carried out in accordance with guidelines of Ethical Committee of NIMS, Japan.

Flow cytometry analysis was used to clarify mechanisms of tumor growth inhibition effect. At the endpoint, mouse was sacrificed, and spleen was collected for analysis. Non-specific staining was prevented by blocking cells with anti-CD16/CD32 antibody (2.4G2, BD Biosciences, USA). Cells were stained with anti-mouse CD_4_ and CD_8α_. Flow cytometry was performed using spectral cell analyzer (Sony, LE-SP6800A). To determine cytokine INF-γ and IL-2 contents, spleen was digested with tissue protein extraction reagent, and analyzed for INF-γ and IL-2 using ELISA system (BD Biosciences).

In addition, at the endpoint, lymph node of mice near the tumor sites were obtained, embedded in paraffin, made section and stained with haematoxylin and eosin (HE) dyes.

## Electronic supplementary material

Supplementary Information
