# Supplementary material for: Hierarchically porous, and Cu- and Zn-containing γ-AlOOH mesostrands as adjuvants for cancer immunotherapy
Source: Sci Rep. 2017 Dec 1;7:16749. doi: 10.1038/s41598-017-12446-9 (PMC5711866; doi:10.1038/s41598-017-12446-9)
Supplement: Supplementary file 1 — Supplementary Information [file 41598_2017_12446_MOESM1_ESM.pdf]

Hierarchically porous, and Cu- and Zn-containing γ-AlOOH mesostrands as adjuvants for cancer immunotherapy

Xia Li[1*], Mohamed A. Shenashen[1], Xiupeng Wang[2], Atsuo Ito[2], Akiyoshi Taniguchi,[3,4] Sherif A. EI-Safty [1,4]*

1 Green Recycling Process Group, Research Center for Functional Materials, National Institute for Materials Science, 1-2-1 Sengen, Tuskuba, Ibaraki 305-0047, Japan
2 Human Technology Research Institute, National Institute of Advanced Industrial Science and Technology (AIST), Central 6, 1-1-1 Higashi, Tsukuba, Ibaraki 305-8566, Japan.
3Cellular Functional Nanomaterials Group, Research Center for Functional Materials, National Institute for Materials Science, 1-1 Namiki, Tsukuba, Ibaraki 305-0044, Japan
4Graduate School of Advanced Science and Engineering, Waseda University, 3-4-1 Okubo, Shinjuku-Ku, Tokyo, 169-8555, Japan

TeL: +81-29-859-2135
FAX: +81-29-859-2501
E-mail: sherif.elsafty@nims.go.jp; sherif@aoni.waseda.jp
E-mail: li.xia@nims.go.jp ; lixia6969a@gmail.com
http://www.nims.go.jp/waseda/en/labo.html
http://www.nano.waseda.ac.jp/

Characterization. The prepared samples were characterized using a JEM-2100 transmission electron microscope (TEM). The crystalline structures were analyzed by X-ray diffractometry using a 18-kW diffractometer (Bruker D8 Advance) at a scan rate of 10°/min with monochromated CuK_{α}-X-radiation (λ = 1.54178 Å). The DIFRAC plus Evaluation Package (EVA) software with the PDF-2 Release 2009 databases provided by Bruker AXS was used to analyze the diffraction and structural analysis data. The TOPAS package program was applied to integrate various types of XRD analyses. The pore structure distribution and surface area were estimated by N_2 adsorption–desorption isotherms at 77 K using a BELSORP36 analyzer (JP. BEL Co., Ltd.). The samples were thermally treated at 120 °C for at least 6 h under N_2 atmosphere. The specific surface area (S_{BET}) was calculated using the Brunauer–Emmett–Teller method with multipoint adsorption data from the linear section of the N_2 adsorption isotherm. The pore size distribution was determined using nonlocal DFT (NLDFT). Zeta potential was analyzed using a zeta-potential and particle size analyzer (ELSZ-1000, Photal, Otsuka electronics) in an appropriate buffer. FTIR spectra were recorded on a FTIR-350 spectrometer (JASCO, Japan) by the KBr pellet method. Particle size distribution of the particles in PBS buffer was analyzed by a dynamic light scattering photometer (DLS-8000HAL, Otsuka Electronics, Japan). Ultraviolet-visible-near infrared (UV-VIS-NIR) spectrophotometer (SolidSpec-3700, Shimadzu) was used to record the OVA

concentration before and after loading.

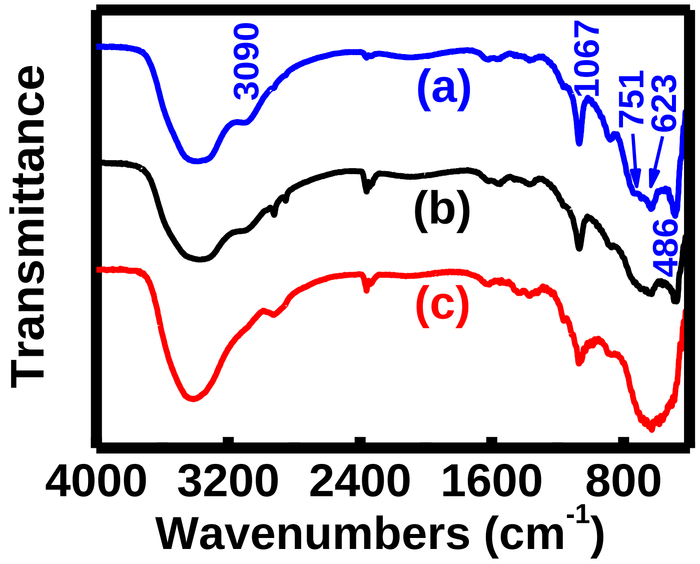

Supplementary Figure 1. FTIR spectra of AMSs (a), Cu-AMSs (b) and Zn-AMSs (c).

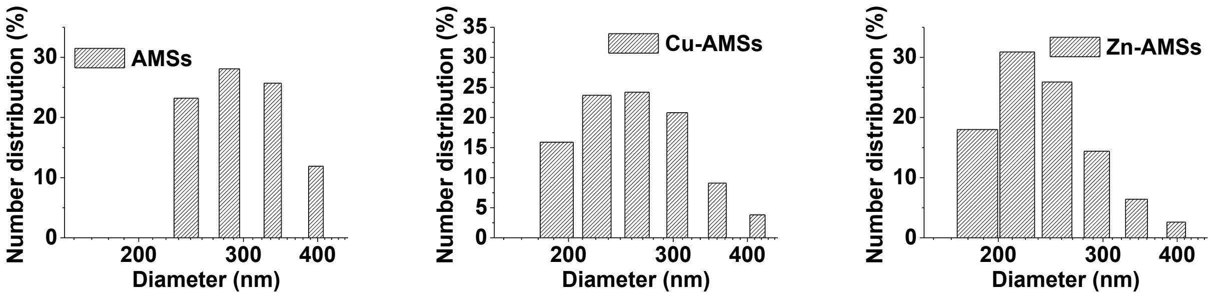

Supplementary Figure 2. Hydrodynamic size distribution of the samples.

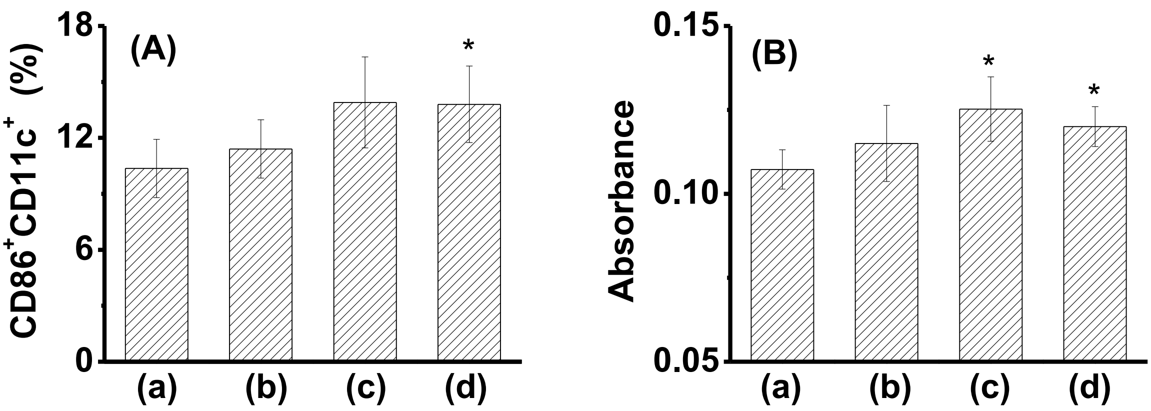

Supplementary Figure 3. BMDCs maturation (A) and INF-γ cytokine release (B) for control (a), AMSs (b), Cu-AMSs (c) and Zn-AMSs (d).

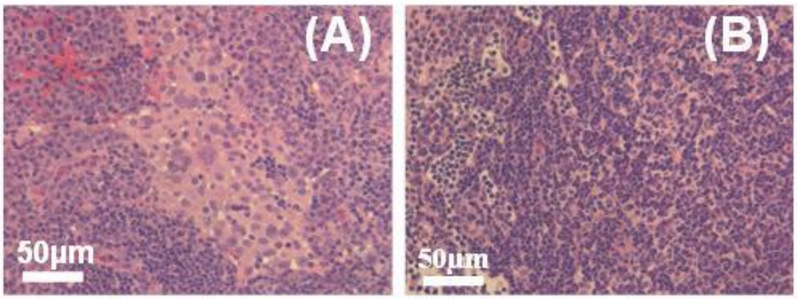

Supplementary Figure 4. Histological sections of lymph node for control (A) and Zn-MASs (B) at the experimental endpoint.
